# Growth Hormone Deficiency in a Child with Neurofibromatosis-Noonan Syndrome

**DOI:** 10.4274/jcrpe.2070

**Published:** 2016-03-01

**Authors:** Doğuş Vurallı, Nazlı Gönç, Dominique Vidaud, Alev Özön, Ayfer Alikaşifoğlu, Nurgün Kandemir

**Affiliations:** 1 Hacettepe University Faculty of Medicine, Department of Pediatric Endocrinology, Ankara, Turkey; 2 EA7331, Université Paris Descartes, Sorbonne Paris Cité, Faculté de Pharmacie de Paris, Paris, France; 3 Service de Biochimie et de Génétique Moléculaire, Hôpital Cochin, Assistance Publique-Hôpitaux de Paris, Paris, France

**Keywords:** Growth hormone deficiency, growth hormone, neurofibromatosis-Noonan syndrome, NF1 gene, neurofibromatosis type 1, Noonan syndrome

## Abstract

Neurofibromatosis-Noonan syndrome (NFNS) is a distinct entity which shows the features of both NF1 (neurofibromatosis 1) and Noonan syndrome (NS). While growth hormone deficiency (GHD) has been relatively frequently identified in NF1 and NS patients, there is limited experience in NFNS cases. The literature includes only one case report of a NFNS patient having GHD and that report primarily focuses on the dermatological lesions that accompany the syndrome and not on growth hormone (GH) treatment. Here, we present a 13-year-old girl who had clinical features of NFNS with a mutation in the NF1 gene. The case is the first NFNS patient reported in the literature who was diagnosed to have GHD and who received GH treatment until reaching final height. The findings in this patient show that short stature is a feature of NFNS and can be caused by GHD. Patients with NFNS who show poor growth should be evaluated for GHD.

**WHAT IS ALREADY KNOWN ON THIS TOPIC?**

Neurofibromatosis-Noonan syndrome (NFNS) is a distinct entity which has variable features of both NF1 (neurofibromatosis 1) and Noonan syndrome (NS). Mutations in the NF1 gene were identified in majority of NFNS cases. Growth hormone deficiency (GHD) has been relatively frequently identified in NF1 and NS, there is limited experience with GHD in NFNS cases.

**WHAT THIS STUDY ADDS?**

Short stature is a feature of NFNS; however, in some cases it can be caused by GHD and patients with NFNS who are not growing sufficiently should be evaluated for GHD. The case presented herein had clinical features of NFNS with a mutation in the NF1 gene. It is the first NFNS case reported in the literature with GHD, receiving growth hormone (GH) treatment, and reaching a successful final height under GH treatment.

## INTRODUCTION

Neurofibromatosis-Noonan syndrome (NFNS, OMIM 601321) was first defined as a distinct entity in 1985 by Allanson et al (1) who reported four unrelated patients with neurofibromatosis (NF) who also had manifestations of Noonan syndrome (NS). These cases had presented with clinical findings such as short stature, ptosis, midfacial hypoplasia, webbed neck, learning disabilities, and muscle weakness (1). Opitz and Weaver (2) also reported a similar syndrome, defined as a separate clinical entity which they named NFNS. This entity bore the features of both NF type 1 (NF1) and NS. These early reports were followed by others (3,4,5,6,7,8,9,10,11). When the genetic studies performed on NFNS are reviewed, it is noted that a mutation was identified in the NF1 gene in the majority of these studies. The co-occurrence of NF1 and PTPN11 mutations has been shown in very few studies and has been attributed to a de novo mutation either in NF1 or PTPN11 gene (12,13). Today, the opinion that NFNS originates from different mutations at distinct genes affecting a common intracellular signal transduction pathway called RAS-MAPK (mitogen-activated protein kinase) pathway is more widely accepted. This pathway plays roles in cell proliferation, differentiation, and apoptosis. The number of affected genes in the RAS-MAPK pathway and the diversity of the mutations in these genes result in various different phenotypic characteristics and different syndromes. Since these syndromes are associated with the effects on the same pathway, they are called “RASopathies” or RAS-MAPK syndromes and NFNS is an important RASopathy.

Growth hormone deficiency (GHD) has been relatively frequently identified in NF1 and NS patients. Those receiving growth hormone (GH) treatment have been published as case reports and the growth pattern, GH responses, near-adult, and adult heights of these cases have been reported (14,15,16,17). However, the literature includes only one study that shows GHD in NFNS cases and that report primarily focuses on the dermatological lesions that accompany NFNS (18). GH treatment in NFNS is still a matter of debate. To our knowledge, the case presented herein is the first reported NFNS patient with GHD who received GH treatment and was followed until she reached final height under GH treatment.

## CASE REPORT

**Figure 1:**
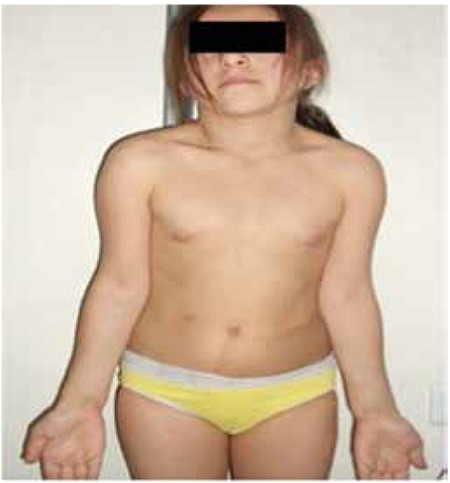
Physical findings of the patient suggestive of both neurofibromatosis 1 and Noonan syndrome

A 13-year-old girl presented with short stature. Physical examination showed dysmorphic facial features, a short and webbed neck, low posterior hairline, cubitus valgus, brachy- and clinodactyly, and widely spaced nipples suggesting NS and multiple café-au-lait spots (>15 mm, 8 spots), axillary freckling, and relative macrocephaly suggesting NF1 syndrome. Dysmorphic facial features included midfacial hypoplasia, prominent nasolabial folds, low-set and posteriorly rotated ears, hypertelorism, downslanted palpebral fissures, and low nasal root (Figure 1). The patient did not have any neurofibroma. Cardiovascular examination revealed no cardiac murmur and echocardiography was normal. The ocular examination did not reveal Lisch nodules. There was no sign of developmental delay, and the nervous system examination was completely normal. The patient’s pubertal stage was evaluated as Tanner stage 2. Her arm span was 124.8 cm and upper/lower ratio was 0.9 suggesting no skeletal deformity. Karyotype analysis was 46,XX. The auxological parameters of the case at diagnosis are given in Table 1.

Complete blood count, routine biochemistry, and urine analysis were within the normal limits. The celiac antibodies were negative and thyroid function tests were normal. Both serum insulin-like growth factor-1 (IGF-1) and IGF binding protein 3 (IGF-BP3) levels were below -3 standard deviation score (SDS). Peak GH response to L-dopa and clonidine stimulation tests were 3.9 ng/mL and 4.2 ng/mL, respectively. Other pituitary hormone levels were all within normal ranges. The serum pituitary hormone levels at diagnosis are given in Table 2.

**Figure 2:**
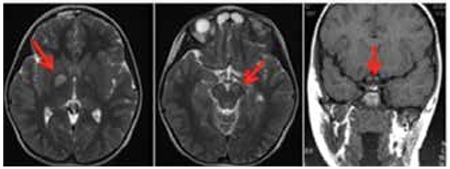
Cranial magnetic resonance imaging of the patient showing the lesions of neurofibromatosis in the cerebral peduncles and globus pallidus and adenopituitary hypoplasia in the pituitary

The size of the pituitary gland was measured as 3.5 mm in the pituitary magnetic resonance imaging (MRI) and this was considered to be consistent with anterior pituitary hypoplasia according to the age group of the patient. NFNS syndrome was suspected, and cranial MRI was performed to evaluate the neurological involvement. Cranial MRI showed a hyperintense mildly swollen appearance in T2 at the cerebral peduncles and globus pallidus that may be attributed to NF. T1-weighed images also showed hyperintense lesions associated with T1 limitation; after administration of intravenous contrast substance, these lesions did not show any uptake of the contrast (Figure 2).

**Figure 3:**
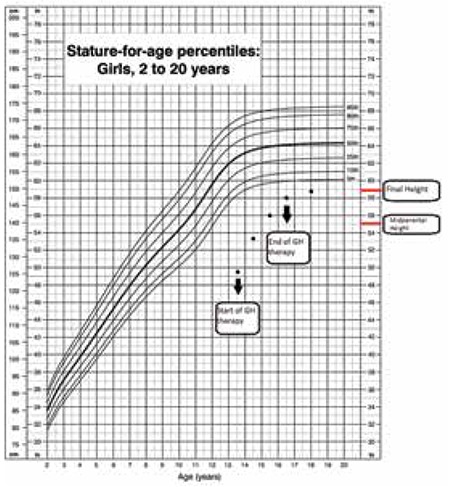
Growth chart of the patient under growth hormone therapy. Source: http:/www.cdc.gov/growth charts (Centers for Disease Control and Prevention (CDC) 2000)

Since the patient fulfilled the criteria of GHD, GH therapy was initiated with a dosage of 0.3 mg/kg/week. The height velocity during the first year of GH treatment was 9.8 cm/year and was 7.2 cm and 4.5 cm on the second and third years of GH therapy, respectively. The patient’s height was 147.3 cm (height SDS: -2.3) at the end of the third year of GH therapy. The patient was 16.5 years old when the GH therapy was discontinued; she had had three regular menstruation cycles and her bone age was 14.5 years. Her final height was 148.5 cm, 7.5 cm above the mid-parental height (Figure 3).

The patient’s father also had features representing both NF1 and NS such as multiple café-au-lait spots, short stature, relative macrocephaly, and axillary freckling, suggesting NF syndrome, and findings such as prominent nasolabial folds, low-set ears, low nasal root, dysmorphic facial features, short neck, and cubitus valgus suggesting NS. It was learned that some members of the father’s family also had multiple café-au-lait spots. Genetic analyses were performed for the patient and her father to investigate NFNS and the genetic analysis of both the patient and the father revealed a truncating mutation c.7846C>T (M82814), p.Arg2616X (AAA59924) in the NF1 gene. No mutation was found in PTPN11 gene.

## DISCUSSION

NFNS is an entity presenting with clinical characteristics of both NF1 and NS. The frequency of NFNS is thought to be higher than the current estimates, since these cases may be missed due to their being inadvertently diagnosed as classic NF1 or NS. In the study of Colley et al (11), the reassessment of 94 cases diagnosed with NF1 has demonstrated that 12 of these cases actually met the criteria for NS. This reassessment has shown that some patients who have been clinically diagnosed with NF1 or NS can indeed be NFNS.

The genetic studies that have been undertaken to identify the gene causing NFNS have shown that the majority of these cases have a mutation in the NF1 gene. These studies have revealed that the mutations responsible for classic NF1 can also cause NFNS (19,20,21). The mutation identified in the present case is indeed a mutation that is seen in classical NF1 cases. Additional studies are required to clarify which mutations cause classic NF1, which mutations cause NFNS, and which mutations have the potential to cause both.

Short stature is a common feature of NFNS as it is of NF1 and NS (22). The frequent causes of short stature in these syndromes are skeletal deformities and nutritional problems. Presence of suprasellar lesions is also a frequent cause, but GHD can develop in some of these patients in the absence of an underlying suprasellar lesion (15). While many studies have reported presence of GHD in NF1 and NS, to our knowledge, there is only one case report on NFNS receiving GH therapy (18). It is known that NS patients are of normal height and weight at birth and that growth deficiency develops later, with almost 80% of the cases eventually being of short stature (23). Several studies have shown that GH treatment increases the final height in NS cases (24,25,26). Among these studies, the one with the highest number of cases is the National Cooperative Growth Study (27). This study involves a large cohort of 252 NS cases who have received GH therapy for 5.6 years on average. GHD can also be seen in NF1 cases in the absence of suprasellar lesions. Some researchers suggested that there could be a relationship between GHD and NF1 in the absence of an organic pituitary damage, and they agree that larger cohort studies are required to decide whether NF1 is a cause of GHD (15,28). GHD-specific screening was recommended in NF1 cases with insufficient growth. An impairment in the cellular signal transduction was suggested as the reason of GHD in NF1 cases without suprasellar regions (29). Hegedus et al (30) showed that neurofibromin provides somatic growth by affecting the hypothalamic-pituitary axis. In their study, body weight and anterior pituitary gland size were found to decrease in mice with an inactivated NF1 gene. It was also shown that the decrease in anterior pituitary size reduces neurofibromin expression in the hypothalamus, thereby decreasing the production of GH-releasing hormone, that of GH and IGF-1 as well.

There is only one case report in the literature about GHD and GH treatment in NFNS. As mentioned above, this one report focuses on the dermatological lesions in NFNS and gives no detailed information about GH treatment. Thus, the present manuscript is first to provide details of GH treatment in a NFNS case. The growth pattern of our patient showed that short stature had been a problem since early childhood, but that the problem had gradually increased within the last 2 years, during which her peers entered puberty and had pubertal growth. There was no underlying reason, such as severe skeletal deformities, suprasellar lesions, or nutritional deficiency to explain the short stature observed in the present case, thus initially, the short stature was considered to be related to the delay in puberty, as in NS cases. At admission, the height of our patient was <-2 SDS and her growth rate was very low. GHD was considered to be a possible reason for short stature, and GH stimulation tests were performed. The peak GH level was calculated as <5 ng/mL in two GH stimulation tests suggesting that the patient had severe GHD. While the final height predicted based on bone age at the beginning of GH therapy was 137 cm, the final height after GH therapy was 148.5 cm. GH therapy resulted in an 11.5 cm (1.8 SDS) gain in the final height.

Recent studies indicate that NS and NF1 patients also benefit from GH treatment. In one study, the height gain based on CDC standards was 8.9 cm for boys and 10.0 cm for girls in NS cases receiving GH treatment and this gain was similar to that in Turner syndrome cases (27). In another study evaluating NF1 cases receiving GH treatment, the growth rate, which was 5 cm/year before GH therapy, increased to 9 cm/year at the first year of therapy, was 8.3 cm/year in the second year, and decreased to 6 cm/year between the third and the fifth years of treatment (15). Although the final height of our patient was 7.5 cm greater than the midparental height, it was still short due to the underlying familial short stature and the relatively short duration of GH therapy. The reason the father had a short stature was likely due to the fact that he also had NFNS, was not assessed with respect to GHD, and did not receive any treatment. When the patient was referred to our clinic, her puberty had already started. A better final height could possibly be achieved if GH treatment could have been started at a younger age. Studies have shown that the earlier that GH therapy is initiated in NF1 and NS cases, the better the final height that can be achieved (14,15).

There is limited experience with GHD in NFNS cases, since it is a rare condition that is clinically difficult to identify. Our patient had the clinical features of NFNS and was found to have a mutation in the NF1 gene. She also had GHD and responded very well to GH treatment. It may be argued that short stature is a feature of NFNS, but it is evident that in some cases, short stature can be caused by GHD. For this reason, patients with NFNS who are not growing sufficiently should be evaluated for GHD. Those diagnosed to have GHD can benefit from GH treatment. However, it is obvious that more studies are needed on the use and benefits of GH therapy in NFNS cases, and also in NF1 and NS cases.

## Ethics

Informed Consent: It was taken.

Peer-review: External peer-reviewed.

## Figures and Tables

**Table 1:**
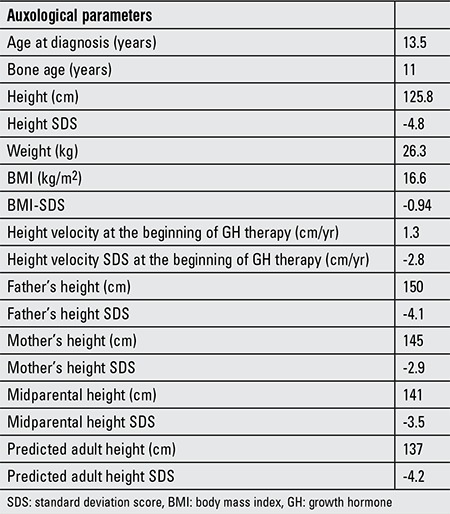
Auxological data of the case at the time of diagnosis

**Table 2:**
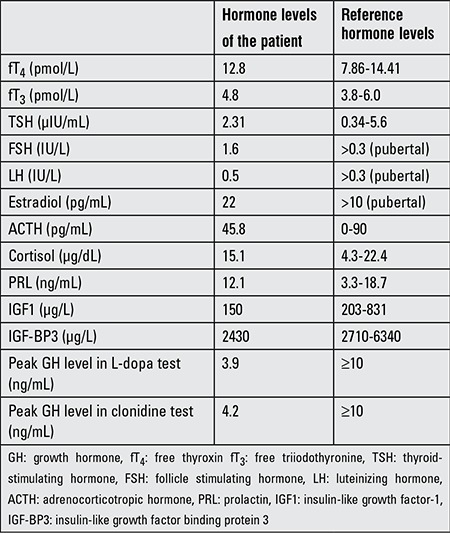
Pituitary hormone levels of the case at diagnosis

## References

[1] Allanson JE, Hall JG, Van Allen MI (1985). Noonan phenotype associated with neurofibromatosis. Am J Med Genet.

[2] Opitz JM, Weaver DD (1985). The neurofibromatosis-Noonan syndrome. Am J Med Genet.

[3] Kaplan P, Rosenblatt B (1985). A distinctive facial appearance in neurofibromatosis von Recklinghausen. Am J Med Genet.

[4] Mendez HM (1985). The neurofibromatosis-Noonan syndrome. Am J Med Genet.

[5] Saul RA (1985). Noonan syndrome in a patient with hyperplasia of the myenteric plexuses and neurofibromatosis. Am J Med Genet.

[6] Meinecke P (1987). Evidence that the “neurofibromatosis-Noonan syndrome” is a variant of von Recklinghausen neurofibromatosis. Am J Med Genet.

[7] Quattrin T, McPherson E, Putnam T (1987). Vertical transmission of the neurofibromatosis/Noonan syndrome. Am J Med Genet.

[8] Shuper A, Mukamel M, Mimouni M, Steinherz R (1987). Noonan’s syndrome and neurofibromatosis. Arch Dis Child.

[9] Abuelo DN, Meryash DL (1988). Neurofibromatosis with fully expressed Noonan syndrome. Am J Med Genet.

[10] Stern HJ, Saal HM, Lee JS, Fain PR, Goldgar DE, Rosenbaum KN, Barker DF (1992). Clinical variability of type 1 neurofibromatosis: is there a neurofibromatosis-Noonan syndrome?. J Med Genet.

[11] Colley A, Donnai D, Evans DG (1996). Neurofibromatosis/Noonan phenotype: a variable feature of type 1 neurofibromatosis. Clin Genet.

[12] Bertola DR, Pereira AC, Passetti F, Oliveira PS, Messiaen L, Gelb BD, Kim CA, Krieger JE (2005). Neurofibromatosis-Noonan syndrome: molecular evidence of the concurrence of both disorders in a patient. Am J Med Genet A.

[13] Thiel C, Wilken M, Zenker M, Sticht H, Fahsold R, Gusek-Schneider GC, Rauch A (2009). Independent NF1 and PTPN11 mutations in a family with neurofibromatosis-Noonan syndrome. Am J Med Genet A.

[14] Noonan JA, Kappelgaard AM (2015). The Efficacy and safety of growth hormone therapy in children with noonan syndrome: a review of the evidence. Horm Res Paediatr.

[15] Vassilopoulou-Sellin R, Klein MJ, Slopis JK (2000). Growth hormone deficiency in children with neurofibromatosis type 1 without suprasellar lesions. Pediatr Neurol.

[16] Carmi D, Shohat M, Metzker A, Dickerman Z (1999). Growth, puberty, and endocrine functions in patients with sporadic or familial neurofibromatosis type 1: a longitudinal study. Pediatrics.

[17] Kirk JM, Betts PR, Butler GE, Donaldson MD, Dunger DB, Johnston DI, Kelnar CJ, Price DA, Wilton P, Group tU (2001). Short stature in Noonan syndrome: response to growth hormone therapy. Arch Dis Child.

[18] Reig I, Boixeda P, Fleta B, Morenoc C, Gamez L, Truchuelo M (2011). Neurofibromatosis-Noonan syndrome: case report and clinicopathogenic review of the Neurofibromatosis-Noonan syndrome and RAS-MAPK pathway. Dermatology Online J.

[19] Baralle D, Mattocks C, Kalidas K, Elmslie F, Whittaker J, Lees M, Ragge N, Patton MA, Winter RM, ffrench-Constant C (2003). Different mutations in the NF1 gene are associated with Neurofibromatosis-Noonan syndrome (NFNS). Am J Med Genet A.

[20] De Luca A, Schirinzi A, Buccino A, Bottillo I, Sinibaldi L, Torrente I, Ciavarella A, Dottorini T, Porciello R, Giustini S, Calvieri S, Dallapiccola B (2004). Novel and recurrent mutations in the NF1 gene in Italian patients with neurofibromatosis type 1. Hum Mutat.

[21] De Luca A1, Bottillo I, Sarkozy A, Carta C, Neri C, Bellacchio E, Schirinzi A, Conti E, Zampino G, Battaglia A, Majore S, Rinaldi MM, Carella M, Marino B, Pizzuti A, Digilio MC, Tartaglia M, Dallapiccola B (2005). NF1 gene mutations represent the major molecular event underlying neurofibromatosis-Noonan syndrome. Am J Hum Genet.

[22] Nyström AM, Ekvall S, Allanson J, Edeby C, Elinder M, Holmström G, Bondeson ML, Annerén G (2009). Noonan syndrome and neurofibromatosis type I in a family with a novel mutation in NF1. Clin Genet.

[23] Mendez HM, Opitz JM (1985). Noonan syndrome: a review. Am J Med Genet.

[24] Osio D, Dahlgren J, Wikland KA, Westphal O (2005). Improved final height with long-term growth hormone treatment in Noonan syndrome. Acta Paediatr.

[25] Municchi G, Pasquino AM, Pucarelli I, Cianfarani S, Passeri F (1995). Growth hormone treatment in Noonan syndrome: report of four cases who reached final height. Horm Res.

[26] Noordam C, Peer PG, Francois I, De Schepper J, Otten BJ (2008). Long-term GH treatment improves adult height in children with Noonan syndrome with and without mutations in protein tyrosine phosphatase, non-receptor-type 11. Eur J Endocrinol.

[27] Romano AA, Dana K, Bakker B, Davis DA, Hunold JJ, Jacobs J, Lippe B (2009). Growth response, near-adult height, and patterns of growth and puberty in patients with noonan syndrome treated with growth hormone. J Clin Endocrinol Metab.

[28] Cnossen MH, Stam EN, Cooiman LC, Simonsz HJ, Stroink H, Oranje AP, Halley DJ, Goede-Bolder A, Niermeijer MF (1997). Endocrinologic disorders and optic pathway gliomas in children with neurofibromatosis type 1. Pediatrics.

[29] Marshall M (1995). Interactions between Ras and Raf: key regulatory proteins in cellular transformation. Mol Reprod Dev.

[30] Hegedus B, Yeh TH, Lee da Y, Emnett RJ, Li J, Gutmann DH (2008). Neurofibromin regulates somatic growth through the hypothalamic-pituitary axis. Hum Mol Genet.

